# The unique pseudanthium of *Actinodium* (Myrtaceae) - morphological reinvestigation and possible regulation by *CYCLOIDEA*-like genes

**DOI:** 10.1186/2041-9139-4-8

**Published:** 2013-03-01

**Authors:** Regine Claßen-Bockhoff, Raili Ruonala, Kester Bull-Hereñu, Neville Marchant, Victor A Albert

**Affiliations:** 1 Institut für Spezielle Botanik und Botanischer Garten, Johannes Gutenberg-Universität, Mainz, 55099, Germany; 2 Department of Biological Sciences, University at Buffalo (SUNY), Buffalo, NY, USA; 3 Kings Park and Botanic Garden, West Perth, WA, 6005, Australia

**Keywords:** Asteraceae, CYCLOIDEA, Gene expression, Inflorescence development, Myrtaceae, Pseudanthium, TCP

## Abstract

**Background:**

Genes encoding TCP transcription factors, such as *CYCLOIDEA*-like (*CYC*-like) genes, are well known actors in the control of plant morphological development, particularly regarding the control of floral symmetry. Despite recent understanding that these genes play a role in establishing the architecture of inflorescences in the sunflower family (Asteraceae), where hundreds of finely organized flowers are arranged to mimic an individual flower, little is known about their function in the development of flower-like inflorescences across diverse phylogenetic groups. Here, we studied the head-like pseudanthium of the Australian swamp daisy *Actinodium cunninghamii* Schau. (Myrtaceae, the myrtle family), which consists of a cluster of fertile flowers surrounded by showy ray-shaped structures, to fully characterize its inflorescence development and to test whether *CYC*-like genes may participate in the control of its daisy-like flowering structures.

**Results:**

We used standard morphological and anatomical methods to analyze *Actinodium* inflorescence development. Furthermore, we isolated *Actinodium CYC*-like genes using degenerate PCR primers, and studied the expression patterns of these genes using quantitative RT-PCR. We found that the ray-shaped elements of *Actinodium* are not single flowers but instead branched short-shoots occasionally bearing flowers. We found differential expression of *CYC*-like genes across the pseudanthium of *Actinodium*, correlating with the showiness and branching pattern of the ray structures.

**Conclusions:**

The *Actinodium* inflorescence represents a novel type of pseudanthium with proximal branches mimicking ray flowers. Expression patterns of *CYC*-like genes are suggestive of participation in the control of pseudanthium development, in a manner analogous to the distantly related Asteraceae. As such, flowering plants appear to have recruited *CYC*-like genes for heteromorphic inflorescence development at least twice during their evolutionary history.

## Background

TCP transcription factors have known functions in cell cycle regulation in angiosperms, leading to differential growth at meristems and in individual organs [1-3]. The acronym TCP stems from the three original members of the gene family, *TEOSINTE BRANCHED1* (*TB1*) of maize, *CYCLOIDEA* (*CYC*), and *PROLIFERATING CELL FACTOR* (*PCF*) of rice [2]. *TB1* is a principal maize domestication gene, having been shown to affect whole-plant architecture by restricting growth of axillary branches in a teosinte ancestor [3]. Genes similar to *CYC*, on the other hand, are associated with floral symmetry in many eudicot lineages [4-12]. CYC-like proteins and close relatives of *TB1* in other plants are members of the ECE clade of TCP factors [13]. Among genes of the *CYC1* (ECE1) clade, control of branching similar to that accomplished by TB1 has been characterized in species such as *Arabidopsis thaliana*(L.) Heynh. [14,15], tomato [16], pea [17,18] and rice [19]. Some of those genes assigned to the *CYC2* (ECE2) lineage appear to have been independently recruited many times over as controls of floral symmetry. CYC2-clade TCPs also participate in the control of flower-type differences in the sunflower/daisy family, Asteraceae [20-23]. The flowering head, or capitulum, of Asteraceae is composed of strongly zygomorphic flowers to the outside (commonly called ray flowers) versus actinomorphic flowers (disc flowers) to the inside, leading to a strong overt appearance to a single flower [24,25]. Such inflorescences that bear different flower types are also referred to as pseudanthia [25-27], in that they “mimic” single flowers both in appearance, and likely, in pollinator attracting function. Thus far, no other example of TCP regulatory control over pseudanthial development has been discovered outside of Asteraceae.

**Figure 1:**
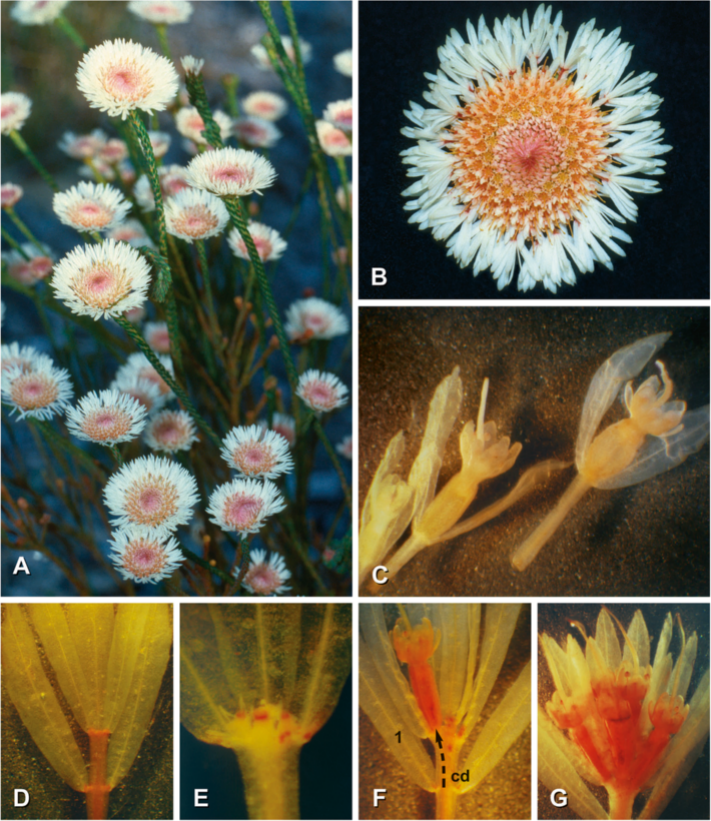
***Actinodium cunninghamii. *A**. The plant grows as an erect shrub with almost unbranched shoots and terminal inflorescences (Cheyne Beach, SW-Australia). **B**. Daisy-like inflorescence with white, ray-shaped elements surrounding the center of fertile flowers (diameter 4 cm). **C**. Fertile flowers illustrating the gradual elongation of the hypopodes from distal (left) to proximal (right) position within the inflorescence. **D-G**. Elements of the showy periphery: **D**, sterile ray; **E**, sterile ray with minute floral buds; **F**, fertile ray with one lateral flower dislocated from the axil of leaf 1 via concaulescence dislocation (cd); **G**, fertile short-shoot with a terminal and four lateral flowers (only three visible).

The Western Australian swamp daisy *Actinodium cunninghamii* Schau. (Figure 1A, B) is characterized by showy, head-like inflorescences that at first glance appear very similar to those of the sunflower family despite the plant being much more closely related to eucalyptus. As in true daisies, a cluster of tubular fertile flowers is surrounded by white, ray-shaped structures. The outer structures were originally interpreted as sterile flowers, with their bracteoles, sepals and petals being modified to showy white elements [28,29]. According to this interpretation the inflorescence is similar to the pseudanthium of daisies. However, recent studies have raised doubts as to this inflorescence interpretation (N. Marchant, unpubl. data), requiring a careful morphological reanalysis. Here, we provide a complete morphological reinterpretation of the *Actinodium* pseudanthium, demonstrating that its structure is completely different from Asteraceae capitula. We also provide evidence that TCPs of the CYC clade are involved in the regulation of *Actinodium* inflorescence structure, and that these proteins are likewise distinct from those operating in Asteraceae, being members of the CYC1 lineage.

## Methods

### Plant material

*Actinodium cunninghamii* is the only species in the monotypic genus *Actinodium* Schau. Its isolated taxonomic position within the Myrtaceae is partly due to its unique inflorescence differing from those of any other Myrtaceae, including those of the related genus *Darwinia* Rudge [30].

For the morphological analysis, plant material was collected in 1985 and 1999 by RCB with permission of the Western Australian Department of Environment and Conservation, Perth, at the Fitzgerald River National Park, 30 km east of Denmark and at Cheyne Beach. Buds and inflorescences were fixed in 70% ethanol for morphological and ontogenetic studies. Vouchers are deposited in the Herbarium of Mainz University (MJG). For the molecular analyses, we used *Actinodium* inflorescences collected in RNAlater (Life Technologies, Grand Island, NY, USA) at Tarrawood Native Nursery (Kalaru, Australia).

### Morphological analysis

For the morphological analysis, flowering plants were investigated in the field and in the laboratory. The number of flowers and rays per inflorescence and of the showy elements per ray were recorded. The rays were carefully investigated and documented by photographs. Histological sections (10 μm) were made with a rotary microtome (Leitz Wetzlar, Germany) according to standard protocols and stained with Toluidine blue. SEM pictures were made by using the ESEM instrument (Philipps Eindhoven, The Netherlands) after critical point drying (BAL-TEC, CPD 030, Leica Microsystems, Buffalo Grove, IL, USA) and sputtering with gold (BAL-TEC, CPD 030, Leica Microsystems, Buffalo Grove, IL, USA) (all according to standard protocols).

### Gene cloning

*Actinodium* genomic DNA was isolated from 60 mg of tissue preserved in RNAlater (Life Technologies, Grand Island, NY, USA) using DNeasy Plant Mini Kit (Qiagen, Valencia, CA, USA) according to the manufacturer’s instructions. To isolate *CYCLOIDEA* (*CYC*)-like genes, degenerate primers designed against TCP and R domains [31] were used in polymerase chain reaction (PCR) amplifications. For each PCR reaction, 25 ng of genomic DNA, 2.5 mM MgCl, 0.15 mM of each dNTP, 0.25 μM of each primer, and 2.5 U of AmpliTaq Gold (Life Technologies, Grand Island, NY, USA) with the provided 1x PCR buffer II (AB) were used in a total volume of 25 μl. Cycling parameters consisted of an initial denaturation for five minutes at 95°C, followed by 35 cycles of 95°C for one minute, 48°C for one minute, 72°C for one minute and a final extension at 72°C for five minutes. PCR products were size-separated on 1.5% agarose gels, and products of the expected sizes were purified using the QIAquick gel extraction kit (Qiagen, Valencia, CA, USA). Selected PCR products were cloned using a PCR cloning kit (Qiagen, Valencia, CA, USA) and PCR-screened using vector primers (M13). Before sequencing, unused dNTPs and primers were removed by ExoSAP-IT (Affymetrix, Santa Clara, CA, USA) treatment. To obtain additional sequence data, a 3^′^ RACE System for Rapid Amplification of cDNA Ends (Life Technologies, Grand Island, NY, USA) and a GenomeWalker Universal Kit (Clontech, Mountain View, CA, USA) were used (see Additional file 1 for primer sequences). To isolate *ACTIN* (*ACT*) genes, we first searched the public database for *ACT* sequences of species belonging to the order Myrtales or Dipsacales. Assuming that these genes are conserved among species, *Syzygium* (GU233755) and *Lonicera* (GQ241342) *ACT* sequences were used to design PCR primers for cloning purposes (*Syzygium*: fwd 5^′^ CAATGTATGTTGCCATTCAG, rev 5^′^ TGGAGTTATATGTGGTCTCGT, *Lonicera*: fwd 5^′^ TTTGCCGGTGATGATGCT, rev 5^′^ ATGTCATCCCAGTTGCTGAC). The subsequent cloning steps were performed as described above. *Actinodium CYC* and *ACT* sequences have been deposited in the GenBank database (accession numbers JQ772501-JQ772505).

### Phylogenetic analysis

Maximum likelihood phylogenetic analysis was performed to gain insight into the relationships among *Actinodium CYC*-like genes. Amino acid sequences from these species and other eudicots were aligned using MUSCLE [32]. Sequences differed considerably outside the highly conserved domains TCP and R domains; therefore, we analyzed only these portions of the corresponding nucleotide alignments (Additional file 2). A single most-optimal tree was computed using the RaxML BlackBox web server (http://phylobench.vital-it.ch/raxml-bb/) running RaxML version 7.2.8 [33]. Default settings were used with the GTR-gamma model of molecular evolution. Accession numbers of the included sequences are provided in the FASTA alignment (Additional file 2). One hundred bootstrap samples were generated to assess support for the inferred relationships. Local bootstrap values (in percentages) are indicated for branches with >50% support.

### Quantitative real-time RT-PCR (qPCR)

For the gene expression analyses, short shoots and flowers within young *Actinodium* inflorescences (diameter approximately 1 cm) were dissected and ground with Lysing matrix A (MP Bio, Santa Ana, CA, USA) in a FastPrep device (MP Bio). Each individual sample consisted of approximately eight short shoots or flowers, including the subtending bracts. Total RNA was isolated using a Spectrum Plant Total RNA kit (Sigma-Aldrich, St. Louis, MO, USA) and treated with on-column DNase as indicated in the manufacturer’s protocol. An iSCRIPT cDNA synthesis kit (Bio-Rad, Hercules, CA, USA) was used to convert 100 ng of the DNase-treated total RNA into cDNA according to the protocol provided by the manufacturer. qPCR was performed using the iQ SYBR Green Supermix ((Bio-Rad, Hercules, CA, USA), 0.1 μM of each primer, and 1/50 of the cDNA template in a MyiQ2 Real-Time PCR Detection system ((Bio-Rad, Hercules, CA, USA), following the recommendations of the manufacturer. Product specificity was evaluated by melting curve analysis. For each sample, the mean of two technical replicates was used as an average value for the threshold cycle (Ct) per individual qPCR experiment. *CYC* expression levels were normalized to *ACT* levels, and relative expression levels were calculated using the formula 2^-∆∆Ct^. The average values of three such independent experiments were determined. Three biological replicates were analyzed, with similar results. The primers used in qPCR experiments are listed in Additional file 1.

## Results

### Morphology and developmental processes

*Actinodium* plants grow as shrubs of 40 to 150 cm in height (Figure 1A, B) in sandy heaths and open forests. Shoots are scarcely branched, densely covered with small closely packed leaves, and terminated by conspicuous head-like inflorescences of 2 to 4 cm in diameter.

**Figure 2:**
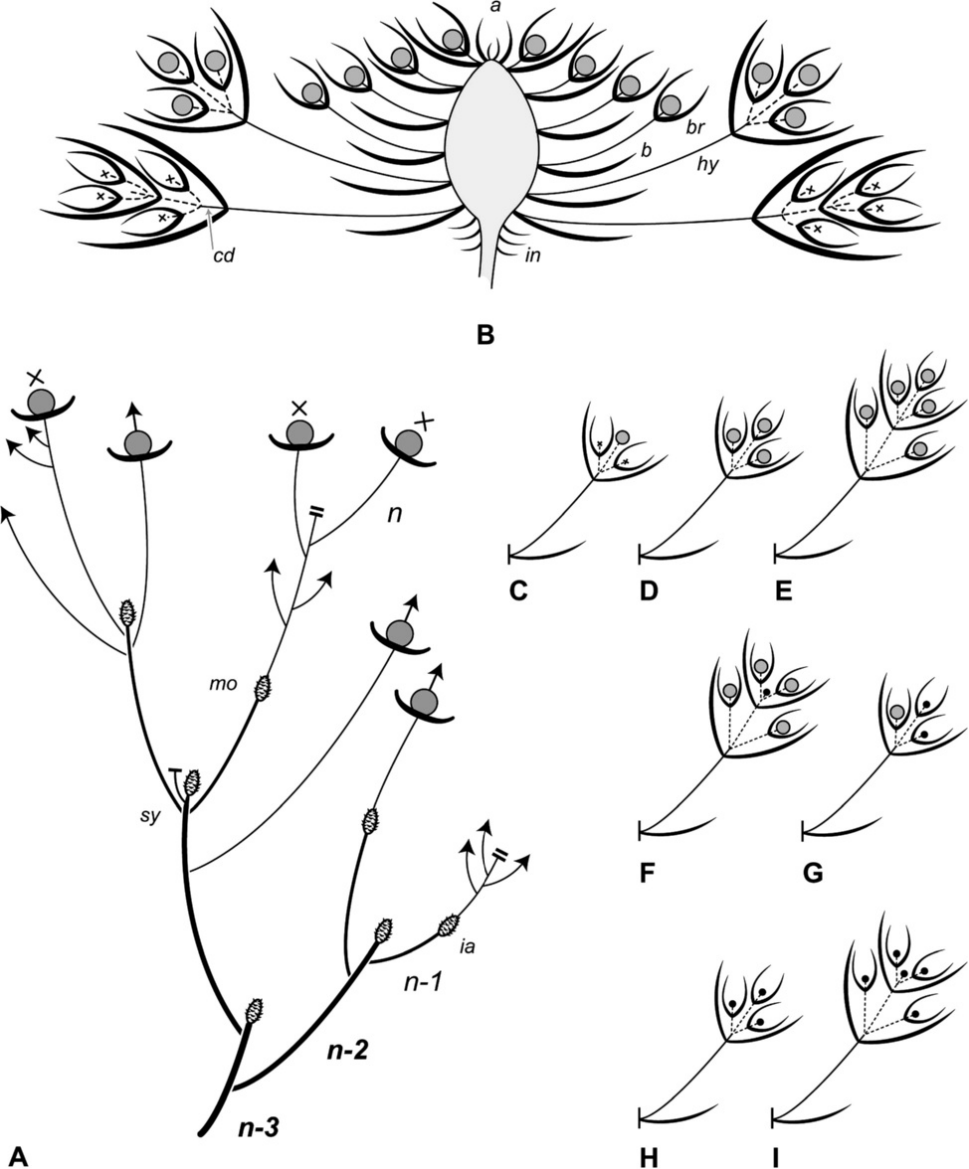
***Actinodium cunninghamii. *A**. Schematic side-view of a four-season old shoot system (n, n-1, n-2, n-3). Dependent on the condition of the apex, the shoot system continues in a sympodial (*sy*) or monopodial (*mo*) manner; the swollen axes indicate former inflorescences (*ia*). **B**. Schematic side-view of the daisy-like inflorescence showing its open apex (a), the cluster of stalked flowers with bracts (b) and bracteoles (br) and the branched short-shoots above the involucre (*in*). Note the concaulescent dislocation (cd), the thickened inflorescence axis and the elongation in hypopode (*hy*) size from distal to proximal thereby arranging the flowers in a plane; **C-I**. Rare elements between the flowers and typical sterile rays (see **B**) with fertile flowers (**C**-**G**) or minute buds (**H**, **I**).

**Figure 3:**
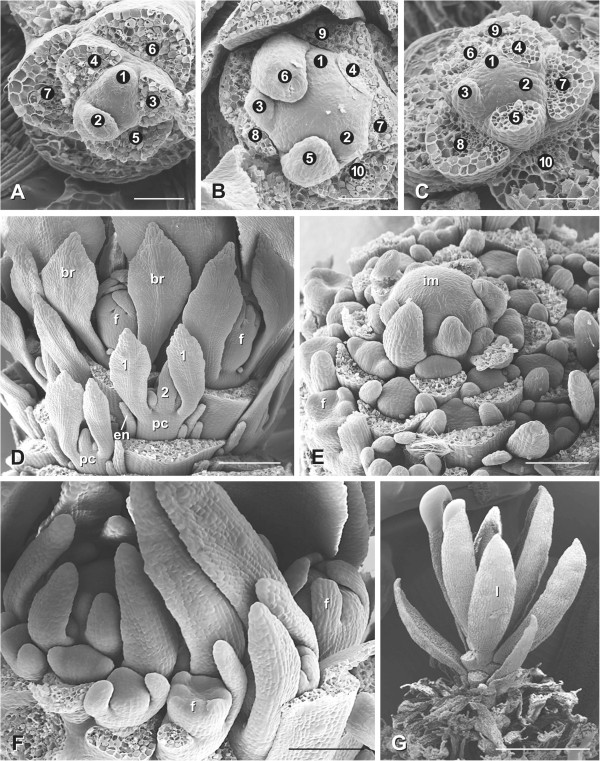
**Development of the inflorescence and paraclades of *Actinodium cunninghamii. *A**. Vegetative meristem before flower production. **B.** Inflorescence meristem with increased size and changed phyllotaxis (two clockwise and three counterclockwise parastichies in **A** and a 3/5 set in **B**). **C**. Vegetative meristem after flower production exhibiting the same phyllotaxis as before flowering. **D**. Side view of a transitional zone of an inflorescence with proximal flower buds (f) and distal paraclades (pc) developing in basipetal sequence. Some bracts have been removed in order to visualize the axillary products. **E**. Inflorescence development showing acropetal flower production. **F**. Tip of an inflorescence primordium producing the last flower buds. **G**. After flower production the meristem continues growth and leaf production (l). 1, 2, leaf pairs at paraclades, br, bracteoles below flower, en, enation, m, meristem. Bars: **A**-**C**: 50 μm; D, 200 μm; **E**, **F**: 100 μm; **G**: 1 mm.

Inflorescences may resume vegetative growth after flowering (Figure 2A: mo) or terminate the shoot, causing sympodial branching pattern on the plant (Figure 2A: sy). In this case, the inflorescence is topped by an aborted vegetative apex (Figure 2B: a). Inflorescences have a flat shape (Figure 1A) and include approximately 100 (49 to 155, n = 21) small, densely aggregated flowers. Each flower has a bract and two whitish, hyaline bracteoles (Figures 1C, 2B: b, br). Some hair-like enations arise beside the bases of the floral hypopodes (Figure 3D: en). While all bracts are of the same size, both the bracteoles and hypopodes become longer towards the periphery of the inflorescence, the latter ranging from almost 0 (distal flowers) to 9 mm (proximal flowers) (Figure 2B).

Inflorescences are surrounded by an average of 17 (8 to 20, rarely up to 41, n = 24) conspicuous rays. Below the rays an involucre is formed by sterile leaves (Figure 2B: in) with broad hyaline margins. Each ray represents a short-shoot bearing an average of 11 (8 to 12, rarely as low as 4 or up to 16; n = 338) white hyaline bracts and bracteoles in a tuft-like arrangement (Figure 1D-G). Usually these shoots show two decussate pairs of bracts each subtending a sterile short shoot with a pair of likewise white and hyaline bracteoles (Figures 1D, 2B). The hypopodes of these short-shoots elongate to a noteworthy length of 5 to 12 mm, the most proximal being the largest ones (Figure 2B: hy). Their second internodes elongate only slightly, thereby dislocating the axillary products of the first bracts in a concaulescent manner (Figures 1D, F, 2B: cd).

The number of nodes and the presence of flowers/floral buds in the ray shoots are variable. These variants are, however, very rare (ca. 7% of all rays investigated; n = 406). Altogether, 15 different forms of ray shoots were found among 29 diverging rays. They include (from proximal to distal) rays with minute reddish structures (Figure 1E), inhibited flower buds (Figure 2H, I), single flowers (Figures 1F, 2C, G) or even up to six well-developed flowers (Figures 1G, 2D-F) in terminal and lateral positions.

Ontogenetic studies indicate that the ray shoots differentiate in a basipetal order showing the hyaline bracts turned towards the main axis (Figure 3D: pc, 1). Contrary to the ray shoots, flowers differentiate in an acropetal way (Figure 3E, F).

**Figure 4:**
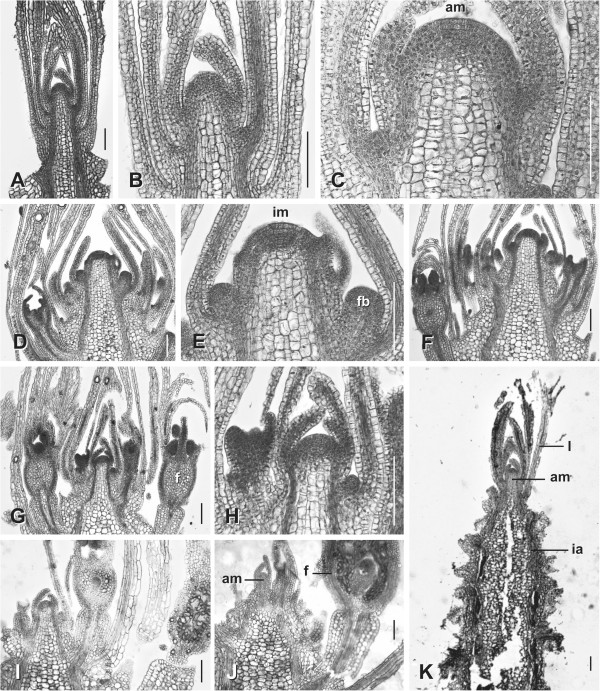
**Histological sections. A-C**. Vegetative shoot tips with leaves, small axillary buds and a central zone at the shoot apical meristem (**C**: am). **D, E** (same specimen). Young inflorescences with bracts, axillary flower buds and a central zone at the tip of the inflorescence meristem (**E**: im). **F-H**. Later stages with swollen medullar parenchyma and unchanged meristem tips. **I-K**., At the end of flower production the meristem returns to the vegetative state. Bars: 100 μm.

The inflorescence meristem (Figures 3B, 4E) differs from the vegetative one (Figures 3A, 4B) in its size (from ca. 50 μm to ca. 100 μm), phyllotaxis (set of parastichies from 2 to 3 to 3 to 5), and organ production (bract and flower instead of leaves, Figure 3E). Despite these differences, both meristems evidence a similar histological composition sharing the so-called central zone, characterized by the presence of large and vacuolated cells (Figure 4C: am, 4E: im).

The flat shape of the adult inflorescence is achieved by a significant thickening of the medullar tissue in its proximal part and by differential hypopode length (Figure 4F, J, K).

Interestingly, at the end of flower production, the inflorescence meristem becomes smaller and approximates the diameter and phyllotactic pattern of the vegetative meristem (Figures 3C; 4J-K). A new vegetative shoot can be formed in the next season from this reduced meristem (Figures 2A: mo; 3G: l; 4K: am).

### Differential expression of CYC-like genes across the *Actinodium* pseudanthium

**Figure 5:**
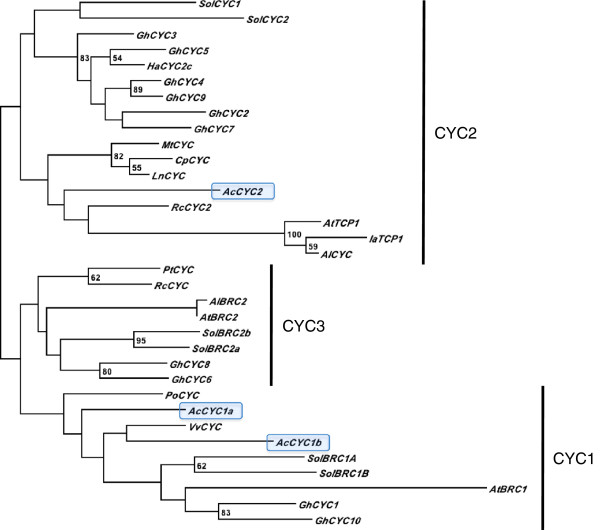
**Phylogenetic analysis of selected *CYC*-like genes.** Analysis is based on nucleotide sequence alignment of TCP and R domains and maximum likelihood analysis. Two of the *Actinodium* (*Ac*) *CYC*-like genes isolated in this study (highlighted in blue) are located in the CYC1-clade, whereas one lies in the CYC2-clade. In addition to *Actinodium*, genes were selected from the following species: *Arabidopsis lyrata* (*Al*), *Arabidopsis thaliana* (*At*), *Cadia purpurea* (*Cp*), *Gerbera hybrida* (*Gh*), *Helianthus annuus* (*Ha*), *Iberis amara* (*Ia*), *Lupinus nanus* (*Ln*), *Medicago truncatula* (*Mt*), *Platanus orientalis* (*Po*), *Populus trichocarpa* (*Pt*), *Ricinus communis* (*Rc*), *Solanum lycopersicum* (*Sol*) and *Vitis vinifera* (*Vv*). Accession numbers are presented in FASTA labels of the nucleotide alignment (Additional file 2). Bootstrap values are shown at nodes.

**Figure 6:**
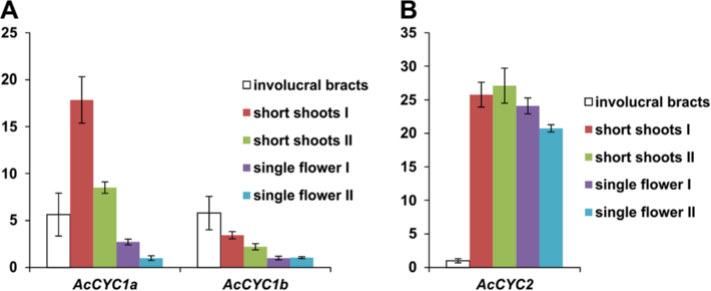
**Quantitative RT-PCR analysis of *Actinodium CYC*-like genes.** Analysis was run throughout the reproductive gradient (colored bars) from outside in (left to right), as well as the involucral bracts (white bars) directly below the pseudanthium. **A**. *AcCYC1a* and *AcCYC1b* (both CYC1-clade genes) show high expression levels in the two outermost ray-like short shoots as compared to inner short-shoots of the pseudanthium. Among the different samples dissected for the study, expression of *AcCYC1a* is highest in the outer short-shoots of the pseudanthium whereas *AcCYC1b* is most prominent in the involucral bracts below them. **B**. *AcCYC2* (aCYC2-clade gene) shows strong expression levels throughout all short-shoots within the pseudanthium, although expression lessens significantly in the innermost shoots. The relative expression levels are normalized against *ACTIN* (accession JQ772504). Bars represent the means of three independent experiments (± standard deviation) including two technical replicates per sample. For each gene, the expression levels are presented as fold differences relative to expression in the lowest expressing sample, which was set to 1. The analyses were repeated using another *ACTIN* sequence (accession JQ772505) for normalization, with similar results.

In an effort to investigate possible molecular mechanisms underlying the inflorescence structure of *Actinodium*, we isolated and studied gene expression levels of *CYC*-like genes in *Actinodium* floral parts. Three *CYC*-like genes were partially amplified in *Actinodium*. Maximum likelihood phylogenetic analysis demonstrated that two of these genes are members of *CYC1* lineage of TCP transcription factors, and one is assigned to the *CYC2* lineage; no *Actinodium* genes of the *CYC3* clade were identified (Figure 5). To evaluate the expression patterns of the *CYC*-like genes, short shoots and flowers in *Actinodium* inflorescences were dissected in a reproductive gradient from outside in. Involucral bracts below the short shoots were included as an additional sample in the study. From qPCR analyses, the two *CYC1*-like paralogs show similarly decreasing expression within the inflorescence from the outside in, but a different expression pattern in the involucral bracts that surround the inflorescence (Figure 6). The *CYC2*-like gene, on the other hand, shows high expression levels throughout the inflorescence, with only a slight decreasing tendency towards the inside.

## Discussion

### The *Actinodium* flowering head is a novel pseudanthium type

Reinvestigation of *Actinodium* inflorescence structure reveals that while *Actinodium* and true daisies correspond in their outward appearance, they represent an exciting example of analogy.

The typical head of a daisy develops from an expanded convex or flattened meristem that produces flowers in a centripetal sequence [34]. Further medullar growth gives rise to the receptacle of the head. The outer flower primordia develop into monosymmetric ray florets while the tubular central florets are polysymmetric. The inflorescence meristem is completely consumed by flower production permitting no further growth, as known for “mantle-core” or “open II-type” inflorescence meristems [35,36].

*Actinodium* differs in at least five basic characters from a true daisy. First, the meristem tip is not involved in the formation of the receptacle. Second, it can proliferate after flowering. Third, it is an inflorescence of the open I-type with a central-zone meristem [35]. Fourth, the rays are not outer flowers, but branched short shoots, which, fifth, originate below the inflorescence and develop in a basipetal order.

In true daisies, the receptacle of the inflorescence originates by the enlargement of the reproductive meristem, which produces sessile flowers in a centripetal order. Meristem expansion is accompanied by a thickening of the medullar tissue that arranges the flowers typically on a flat plane. In *Actinodium*, however, the medullar tissue thickens after flower production, that is, below the active meristem, dislocating the already segregated flowers towards a horizontal plane.

The inflorescence shoot of *Actinodium* is able to continue vegetative growth after flowering, a phenomenon which has been termed by Troll [37] “inflorescence proliferation” (see [38]). This fact led Briggs and Johnson [29] to infer a “flexible condition at the meristem tip” of the *Actinodium* inflorescence (“conflorescence” after their terminology). Nevertheless, there are two possible explanations for the existence of proliferating inflorescences: either they actually rely on an inflorescence meristem that can be reverted to resume vegetative growth after flowering [39-43], or the supposed inflorescence is rather a vegetative shoot bearing lateral reproductive units, thus masking the appearance of a true inflorescence. This can be found in the same family, Myrtaceae (for example, *Callistemon*, *Melaleuca*[44], but also in other ones throughout the angiosperms (*Drimys winteri*[45], *Mahonia aquifolium*, and *Lysimachia nummularia*[44]). Our observation of the sequential transformation of the inflorescence meristem in *Actinodium* definitively fits with the first interpretation.

The capacity of *Actinodium* to resume vegetative growth after flowering surely relies on the maintenance of the central zone in the inflorescence meristem throughout. Comparative developmental studies in open inflorescences have termed these inflorescences as open I [35,46], in contrast to the meristematic organization of daisy heads, which do not show either central zone or proliferation capacity in the wild type at least.

In some Asteraceae, the dense cluster of flowers is surrounded by a circle of ray flowers. These outer flowers differ from the actinomorphic bisexual flowers in the center by their increased corolla size, monosymmetry, and female or sterile nature [24]. Troll [25] termed the flower-like inflorescences pseudanthia (although this term was used in a different context before, see [27]), indicating their overt similarity with flowers to be a classical example of analogy. The remarkable similarity of the swamp daisy *Actinodium cunninghamii* to a true daisy almost certainly prompted Bentham [28] to interpret the rays as sterile flowers. Briggs and Johnson [29], Holm [47] and Claßen-Bockhoff [48] followed his interpretation, and only N. Marchant (unpubl. data), while preparing a revision of the *Chamelaucium* group, found the rare flowers in the ray structures and consequently concluded these to be short-shoots. Ray florets in true daisies are the outermost flowers of the inflorescence. They may originate with some delay compared to the disc florets but always arise from the same head meristem [34,49]. In *Actinodium*, however, such a head meristem does not exist. Experiments in *Arabidopsis thaliana*[50] illustrated vegetative buds below the main inflorescence to be stimulated by light and auxin flow to develop late lateral inflorescences in a basipetal sequence. The ray shoots in *Actinodium* develop in a basipetal order as well, showing concordance with the lateral inflorescences found in *Arabidopsis*. Basipetally flowering shoots separate from the terminal inflorescence have been termed “paraclades” [51] and may well represent the ray shoots in *Actinodium*.

The unique organization of the flower-like inflorescence of *Actinodium*, not known from any other plant family, requires the recognition of a novel pseudanthium type [26,48]. This floral mimic is characterized by an inflorescence meristem with a persistent central zone, able to proliferate, a receptacle originating from medullar thickening, and showy paraclades composed of branched short-shoots.

### The inflorescence of *Actinodium* is influenced by *CYCLOIDEA*-like gene activity

In angiosperms, shoot branching patterns are strictly controlled in order to achieve proper architecture. For example, in *Arabidopsis* and tomato, TCP transcription factors belonging to *CYC1* clade play a key role in arresting axillary bud growth [14-16]. *BRANCHED1* (*BRC1*) in *Arabidopsis*[14,15], and two *BRC1* paralogs in tomato [16] are all expressed in arrested axillary buds and down-regulated upon bud outgrowth. In *Arabidopsis*, the outgrowth of axillary buds typically occurs when the plant transforms to reproductive stage. Similar to tomato, we found two *CYC1*-like genes in *Actinodium* (Figure 5), indicating that gene duplication may have occurred during molecular evolution of both species. The two *Actinodium CYC1*-like genes share an expression pattern that correlates with the branching pattern of short shoots in the pseudanthium (Figure 6). In the outermost elements where the expression level is highest, activity of these genes may contribute to reproductive repression by preventing outgrowth of tiny buds located in the axils of the short shoots. In the inner, non-branched fertile units, the expression levels of *CYC1*-like genes were extremely low. *In situ* analyses of expression patterns in short shoots and flowers were not successful enough to provide tissue-specific expression patterning. Nevertheless, our qRT-PCR data provide strong correlative evidence, although functional studies would be required to confirm a role of *AcCYC1*-like genes in reproductive repression in *Actinodium*. For a non-model organism such as *Actinodium*, use of heterologous systems would be needed, in which case interpretation of results might be challenging. If the *Actinodium* pseudanthium can be compared to an individual *Arabidopsis* plant, with the showy sterile short shoots and their suppressed bud outgrowth analogous to an *Arabidopsis* rosette in its vegetative form, *AcCYC1a/b* and *AtBRC1* might share a function in controlling reproductive development via shoot branch suppression.

While *AtBRC1* seems to have a distinct function in controlling *Arabidopsis* shoot branching, the role of *AtTCP1*, a CYC2-clade gene, has been less clear. Recently, however, *AtTCP1* has been shown to affect shoot development in terms of elongation of leaves, petioles and inflorescence stems [52], possibly in concert with hormonal regulation [53]. In *Actinodium* the slight decrease in expression levels of *AcCYC2* towards the inside of pseudanthium (significantly lower, however, in the innermost short-shoots) correlates with decreased length of bracteoles, and decreased elongation of hypopodes, which contributes to the flat shape of pseudanthium. Thus, in the case of *Actinodium*, a *CYC2*-clade gene may have been recruited to enhance showiness of the inflorescence by bracteole elongation, instead of floral symmetry changes as in the case of Asteraceae. Both strategies may serve in pollinator attraction rather than reproduction, as the ray (or ray-like) elements are often sterile.

In summary, *CYC*-like genes may be involved in providing the *Actinodium* pseudanthium with its unique structure: *AcCYC1a/b* via short-shoot branching and *AcCYC2* via bracteole and hypopode elongation, thereby contributing to the showiness and reproductive success of the inflorescence. Future attempts to clone and characterize the expression of *Actinodium* CYC3-like genes may be similarly illuminating, as at least one such gene has been implicated in the control of flower type in the sunflower [22].

## Conclusions

The *Actinodium* inflorescence represents a novel type of pseudanthium with proximal branches mimicking ray flowers. Expression patterns of *CYC*-like genes are suggestive of participation in the control of pseudanthium development, in a manner analogous to the distantly related Asteraceae. As such, flowering plants appear to have recruited *CYC*-like genes for heteromorphic inflorescence development at least twice during their evolutionary history.

## Abbreviations

ACT: ACTIN; Ct: Threshold cycle; CYC: CYCLOIDEA; PCF: PROLIFERATING CELL FACTOR; TB1: TEOSINTE BRANCHED1.

## Competing interests

The authors declare no competing interests.

## Authors’ contributions

VAA and RC-B designed the research. RC-B supervised the morphological studies, which were executed by her and KB-U. NM provided morphological interpretations. VAA supervised the molecular developmental research, which was executed by RR. VAA, RC-B and RR wrote the paper. All authors read and approved the final manuscript.

## Supplementary Material

Additional file 1 **Primer sequences used for GenomeWalker, 3**^**′ **^**RACE, and quantitative RT-PCR (qPCR) experiments.** Click here for file

Additional file 2 **Nucleotide sequence alignment of selected set of *CYC*-like genes used to reconstruct the phylogenetic tree shown in Figure 5.** GenBank accession numbers for each sequence are included in the FASTA identifiers. Click here for file
