# Supplementary material for: The unique pseudanthium of Actinodium (Myrtaceae) - morphological reinvestigation and possible regulation by CYCLOIDEA-like genes
Source: EvoDevo. 2013 Mar 1;4:8. doi: 10.1186/2041-9139-4-8 (PMC3610234; doi:10.1186/2041-9139-4-8)
Supplement: Additional file 1 — Primer sequences used for GenomeWalker, 3′ RACE, and quantitative RT-PCR (qPCR) experiments. [file 2041-9139-4-8-S1.doc]

| Gene | Accession | Oligo sequence 5’->3’ | Experiment |
| --- | --- | --- | --- |
| *AcCYC1a* | JQ772502 | rev1 GCCGAGTACTGATCCCCTTTACCAGTTG | GenomeWalker |
| *AcCYC1a* | JQ772502 | rev2 GGCAGTGCAACTGTTGTTCATCTTAGCA | GenomeWalker |
| *AcCYC1b* | JQ772503 | rev1 TTCGCCTATTTCAGGCATCATCACTTC | GenomeWalker |
| *AcCYC1b* | JQ772503 | rev2 ATGGCAGGTGTGGATTTCACTAGCAAC | GenomeWalker |
| *AcCYC2* | JQ772501 | rev1 CTCTAGGGTTTTGCTTGCCTTGTCGAAC | GenomeWalker |
| *AcCYC2* | JQ772501 | rev2 CTAGCATGTCCTGGAGGTCGAAGAACCT | GenomeWalker |
| *AcCYC1a* | JQ772502 | fwd1 AGAGCTTTCCAGGAGCATTG | 3’RACE |
| *AcCYC1a* | JQ772502 | fwd2 GCAACTGGTAAAGGGGATCA | 3’RACE |
| *AcCYC2* | JQ772501 | fwd1 CCGATGATGAGAAGGGTTGT | 3’RACE |
| *AcCYC2* | JQ772501 | fwd2 CGATCAGCTGGTGACGACTA | 3’RACE |
| *AcCYC1a* | JQ772502 | fwd GCAACTGGTAAAGGGGATCA | qPCR |
| *AcCYC1a* | JQ772502 | rev AACGAATTCCTGTGGGATTG | qPCR |
| *AcCYC1b* | JQ772503 | fwd ACAACAGCGAAAGCATCCAT | qPCR |
| *AcCYC1b* | JQ772503 | rev TCAGCGATTTCGCCTATTTC | qPCR |
| *AcCYC2* | JQ772501 | fwd CGATCAGCTGGTGACGACTA | qPCR |
| *AcCYC2* | JQ772501 | rev ACATCTCTGCAGGGTGTTCA | qPCR |
| *AcACT* | JQ772504 | fwd TGCTGTGTTTCCCAGTATCG | qPCR |
| *AcACT* | JQ772504 | rev GATTGAGCTTCATCCCCAAC | qPCR |
| *AcACT** | JQ772505 | fwd CCCTCGACTATGAGCAGGAG | qPCR |
| *AcACT** | JQ772505 | rev GGCACCGATTGTAATCACCT | qPCR |
|  |  |  |  |

*Using this set of primers for normalization reproduced the results shown in Fig 6.
