# Supplementary material for: The unique pseudanthium of Actinodium (Myrtaceae) - morphological reinvestigation and possible regulation by CYCLOIDEA-like genes
Source: EvoDevo. 2013 Mar 1;4:8. doi: 10.1186/2041-9139-4-8 (PMC3610234; doi:10.1186/2041-9139-4-8)
Supplement: Additional file 2 — Nucleotide sequence alignment of selected set of CYC-like genes used to reconstruct the phylogenetic tree shown in Figure 5. GenBank accession numbers for each sequence are included in the FASTA identifiers. [file 2041-9139-4-8-S2.doc]

>AtBRC1 NM112741

------------------------------------------------------------

------------------------------------------------------------

------------------------------------------------------------

------------------------------------------------------------

------------------------------------------------------------

------------------------------------------------------------

------------------------------------------------AGAACGGACAGG

---CACAGCAAGATCAAAACGGCCAAAGGGACACGAGATCGTAGGATGAGACTCTCGCTA

GATGTCGCCAAAGAGTTGTTTGGCTTACAAGACATGCTTGGATTTGACAAAGCCAGCAAA

ACCGTTGAATGGTTGCTTACACAAGCAAAACCTGAGATCATAAAGATC------------

------------------------------------------------------------

------------------------------------------------------------

------------------------------------------------------------

---------------------------------------------------AAG---GAG

GAGAGAGCGAAAGC---TAGAGAAAGAGCAAAGGGTAGAACAATGGAGAAA---------

------------------------------------------------------------

------------------------------------------------------------

------------------------------------------------------------

------------------------------------------------------------

------------------------------------------------------------

------------------------------------------------------------

------------------------------------

>IaTCP1 EU145779

------------------------------------------------------------

------------------------------------------------------------

------------------------------------------------------------

------------------------------------------------------------

------------------------------------------------------------

------------------------------------------------------------

------------------------------------------------AAGAAAGACAGG

---CATAGCAAGATTCACACAGCACAAGGGCTTAGAGACAGGAGGGTAAGGCTTTCTATT

GGTATTGCTCGCCAGTTCTTTGATCTCCAGGATATGTTAGGGTTTGATAAAGCCAGTAAA

ACTTTAGACTGGCTACTCAAGAAATCAAGAAAAGCCATCAAAGAGCTT------------

------------------------------------------------------------

------------------------------------------------------------

------------------------------------------------------------

---------------------------------------------------AAG---AGA

TCAAAAGCCAAAGC---T---GAAAAAGCAAAGGAGATGATTTATAAACAT---------

------------------------------------------------------------

------------------------------------------------------------

------------------------------------------------------------

------------------------------------------------------------

------------------------------------------------------------

------------------------------------------------------------

------------------------------------

>AtTCP1 NM001160982

------------------------------------------------------------

------------------------------------------------------------

------------------------------------------------------------

------------------------------------------------------------

------------------------------------------------------------

------------------------------------------------------------

------------------------------------------------AAGAAGGACCGA

---CATAGCAAGATTCAAACGGCACAAGGGATTAGAGACAGGAGGGTTAGGCTTTCTATT

GGGATTGCTCGCCAATTCTTTGATCTTCAGGATATGTTGGGGTTTGATAAAGCTAGTAAA

ACGTTAGACTGGCTGCTCAAGAAGTCAAGAAAAGCCATCAAAGAGGTC------------

------------------------------------------------------------

------------------------------------------------------------

------------------------------------------------------------

---------------------------------------------------AAG---GGG

CTAGGAGCCAAAGC---TAGAGGAAAAGCAAAGGAGCGAACAAAAGAGATG---------

------------------------------------------------------------

------------------------------------------------------------

------------------------------------------------------------

------------------------------------------------------------

------------------------------------------------------------

------------------------------------------------------------

------------------------------------

>AlCYC XM002888537

------------------------------------------------------------

------------------------------------------------------------

------------------------------------------------------------

------------------------------------------------------------

------------------------------------------------------------

------------------------------------------------------------

------------------------------------------------AAGAAGGACCGA

---CATAGCAAGATTCACACGGCACAAGGGCTTAGAGACAGGAGGGTTAGGCTTTCTATT

GGGATTGCTCGCCAATTCTTCGATCTTCAGGATATGTTGGGGTTTGATAAAGCCAGTAAA

ACGTTAGAATGGCTGCTCAATAAATCAAGAAAAGCCATCAAAGAGGTC------------

------------------------------------------------------------

------------------------------------------------------------

------------------------------------------------------------

---------------------------------------------------GAG---GGA

TCAAGAGCCAAAGC---TAGAGGAAAAGCAAAGGAGATGGCCTATGACCAT---------

------------------------------------------------------------

------------------------------------------------------------

------------------------------------------------------------

------------------------------------------------------------

------------------------------------------------------------

------------------------------------------------------------

------------------------------------

>SolCYC2

ATGTTCTCTGCCAGTAATAGTAGTACTCATGATAACCCTCTTCCTCACTACATTTCCTCA

TCCTTTCACACCTCTTCTCCCTTTCTTGGTTTCACTGGCAACCAAATTCTCCTTCATCAA

TATTACCAAAATCAATTTTCTAGTCATTATTACTTGGCAAAGAACAATGAAGATTATTGT

GATAATTCCTTAAGGTCGTTCCCCATGAAGAAGAAATCCAAG------------------

------------------------------------------------------------

------------------------------------------------------------

------------------------------------------------AAAAGGGAGAGG

TCATGTGGTAAGATTTTGACGGCTCAAGGTCCAAGAGATAGAAGGATAAGACTCTCCATT

AACATGGCTAGAAAGTTCTTTGATCTTCAAGAACTTCTAGGTTTTGACAAACCAAGCAAA

ACCATTGATTGGCTATTTACACACTCCGAATTAGCCCTTGAGGAGCTC------------

------------------------------------ACTAATTGCCAATTAGAAACTAAG

ATGTTTTTTGTATGGTCCGTTCAACTAAAATTATTTCTATTCTATT--------------

----------------------------------------ATATTTATGTAGGAGCAATC

AACAACAAGGGTTTAGAAAGAAATCCCAAAAGAGCAAAAGAAGTAACACTAAAA---GAG

TTAAGGAAAAAGGC---AAGAGCACGAGCTAGGGAAAGAACAATCAAGAAAATGTGGACC

AAAATTGAAACTAGCCACAAATCAGCTAATTTTTTTGGAAAAAAAGATATTAGTGAAATG

GAACAACATTTTTTCAAGAACAAATTACAAGCAAACAAAGAAATAATTGAGGGATCTGGA

GTTACAAAAATTAAGATAATGCCTTCTTTAATCTTGGGTTTTAACCCTAATCCTTATGCT

CCAATAGAGTCAGGTACCTACTATGGTGGCTCTTCCTTATCACAAGGTATCGTTTTAGAA

TTTAAGTTATATATACTGAACATTTTCTTTCTTACACTGATCGATGTAATTTAA------

------------------------------------------------------------

------------------------------------

>SolBRC1B

ATGTATCCTCCAAGCAACAATAACTGCAGCCCAATTTTGTCTTCTTTGATATGCCAAAAT

ATTCCATCTTCTCCTTGTATGCAATATGAACACGAACTATACTTTCAAAGCTTTAATCAT

GATAACCAATATTATTTTCAACAACAGCAACTAGTTCCCTCGATAGATGATTTGAGTCCT

CACATCTTAGCTGACAGCTGCACCGAGATTATTACTAAGCCTTCGAATTGCAACCACGAA

CTACAAGGAATGGAAGAAGGCCGAGGCGAAAAGAAAGGAGATGATGATGTTATGAGTAGC

AGAATTAGTGGACGGATCTCAAAAAATAATAAGAGATCTTCC------------------

------------------------------------------------AATAAAGATCGA

---CACAGCAAGATCAACACCGCTCGTGGTCCAAGAGATCGAAGGATGAGACTTTCACTT

GATGCTGCTCGCAAGTTTTTCCGTTTGCAGGACTTATTGGGATTCGATAAGGCCAGCAAA

ACTGTTGAATGGTTGCTTACTCAATCGGATTCTGCAATTGAAGAGCTTGTTGCCGCTAAA

GGCAATGATGCACAGGTTGCTCAGCAAACTAGCTGCAATACCCCCACTACTACTACTGGA

ATTGGTGCAATTTGTGCATCTAATTCTATTTCTGAGTCGTGTGAAGTTATATCAGGAACT

GATGAAACTTCCTCTAATGACAAAAACAAGGAAACCGCTCAAGATGAGGAGAAGAAGAAA

AGGAAGAAGGTGGTTAACACAGCTCGTAGAGCTGTGTTAGAACCTCTTACGAAG---GAA

TCGAGGAATCAAGC---AAGAGCCAGGGCTAGAGAGAGAACAAAATCAAAGAAAATGAGC

CAAACTGGAAAATCCAAATCCCTAGCTAATGATTTGAACCCTTCAGGATCTCGGAGGCCG

GCTAATAAAACTTGTGAAGAACCTGGAACACATGAAGAACTCAACTTCCATCAAGAGAAG

AACACTGTCGATGACTGTAATTTTATGGTAAATGGAAATTGGAATCCATTTACAATCTTT

AGCTATCATGAGCAATACGCTGGAATTTCCAACGAGGTGAGGGTTTCAGACTTTGTTTTT

TAGGGCTTCAATAATTGAACCCACATATTCTTCTCATCTTCTGATTATTATTTTTTTTAA

AAAAAAAAAAATTCTTGTTTCTCTGCAGCATCAATTGGTTACAGACTTGCAATTTTGTGG

AAAGCTATGGGAAGGCTAG-----------------

>GhCYC1 EU429302

------------------------------------------------------------

------------------------------------------------------------

------------------------------------------------------------

------------------------------------------------------------

------------------------------------------------------------

------------------------------------------------------------

------------------------------------------------AAGAAAGATCGT

---CACAGCAAGATTGACACTGCTCGAGGTCCAAGGGACCGAAGGATGAGATTGTCGCTT

GATGTTGCTAAGCAGTTCTTCAGGTTGCAGGACATGCTGGGGTTCGATAAGGCCAGCAAC

ACTGTCGAGTGGCTGCTCATGAAATCAAAAGCCGCTATTCACTATCTC------------

------------------------------------------------------------

------------------------------------------------------------

------------------------------------------------------------

---------------------------------------------------AAA---GAA

ACAAGGGAAAGGGC---AAGAGCAAGGGCAAGGAAGAGGACAAATGAAAAA---------

------------------------------------------------------------

------------------------------------------------------------

------------------------------------------------------------

------------------------------------------------------------

------------------------------------------------------------

------------------------------------------------------------

------------------------------------

>GhCYC10 JN190064

------------------------------------------------------------

------------------------------------------------------------

------------------------------------------------------------

------------------------------------------------------------

------------------------------------------------------------

------------------------------------------------------------

------------------------------------------------AAAAGAGACCGA

---CACAGCAAGATCAACACGGCTCGAGGCCCCAGGGACAGAAGGATGCGATTGTCTCTT

GATGTTGCTAAGAAGTTGTTTGGTTTGCAAGACCTTTTGCGGTTCGATAAGGCTAGCAAG

ACCATTGATTGGCTGATAACGAAATCAAAAACCGCCATTCAAGAACTC------------

------------------------------------------------------------

------------------------------------------------------------

------------------------------------------------------------

---------------------------------------------------AAA---GCA

ACAAGGGAAAGAGC---AAGAGCAAGGGCAAGAGAGAGAACAGTGGAAAAG---------

------------------------------------------------------------

------------------------------------------------------------

------------------------------------------------------------

------------------------------------------------------------

------------------------------------------------------------

------------------------------------------------------------

------------------------------------

>SolBRC1A

ATGTACCCTTCGAGCAATTACAGCCCCAATATTTCCAGCTCTTCATCTTTCTTTCACATT

AATATTCCATCTCCTTCTAGCAATATGAACCCGAATTCATCCAATATTTCCATGATTTTC

AATTCATCCAACCTAGTTACGATCAGAATACCAATATTCCTGCAGAAGAAGCTGCTGATT

CGGACAAACTAGATAAAATAGAAGAAGATCAATCAATCATAAAAAGCTGCAATAATAACA

AGAAGGATGAGAAGAGTAGTAGCAGTACTAGTACTATTCGTAGAAAAAACAACAAGAGAA

CTACGAGTGGTAGTGCTGGTGTAGGACCTTCG----------------------------

------------------------------------------------AAGAAAGATAGA

---CACAGCAAAATCAACACGGCACATGGCCCAAGAGACCGAAGAATGAGACTATCACTT

GAAATTGCTCGCAAATTCTTCAATTTGCAAGACTTGCTTGGGTTCGATAAAGCCAGCAAA

ACTGTAGAATGGCTACTCACAAAGTCAAAATCAGCGGTGAACGATCTGGTTCAGAAAATT

AACAAAGACAAATGCAGCGGTAGTGAAAATCCTAATATTGCTACTGTATCATCTCCTTCC

GCCGAATCATGTGAAGTTATCGACGAATCAGCTGCAACTAATACAG--------------

----------------------------------------CAGAAACTCAGAAGCAACAG

AAGAAAAAAGTTAAGTCGATTCGTAGGGCAATAATTCATCCAGTTGTTGCAAAG---GAA

TCAAGGAAAGAAGC---AAGAGCAAGGGCAAGGGAAAGAACAATAATAAAG---------

---AAAAGCCTAAATGATAACACGAATAATAATAATAATGGTGATCAATCTATGGCTGAT

GAGGATTTAACAAGATCATTAAGATCTTGGAATACTACATTTGAAGATCATCAATCAGGT

ATTCAAGGCTATAATAATAATAATAATATGAATGTTGTTGATAACTTTAATTTGGTGGAT

ACTAGCAATTGGAGCCCATTTATGTTCAACTATCACCAAATCAATACTGAAATTTCTCAA

GAGGTATGTACTAATTTAATTAATAAATTATTTTTTCTATTATTATTATTAACCCGATCG

CCAAGTATTTATTTATATTTTTGTGTTGCAGCATCAATTTGCGAACTTCCAGTATTCTGG

GAAGTTATGGGAAGCTTAATTAG-------------

>AcCYC1b JQ772503

------------------------------------------------------------

------------------------------------------------------------

------------------------------------------------------------

------------------------------------------------------------

------------------------------------------------------------

------------------------------------------------------------

------------------------------------------------AAGAAAGATCGG

---CACAGCAAGATTAACACCGCTCAAGGCATGAGAGATCGGAGGATGAGGTTGTCCGTT

GAAGTCGCTCGCGAATTCTTCAATCTTCAAGACATGCTCGGTGTCGACAAGGCTAGCAAA

ACCATCAAGTGGTTGCTAGTGAAATCCACACCTGCCATCAAAGAATTA------------

------------------------------------------------------------

------------------------------------------------------------

------------------------------------------------------------

---------------------------------------------------GGA---GAG

TCGAGGGCGA--------------------------------------------------

------------------------------------------------------------

------------------------------------------------------------

------------------------------------------------------------

------------------------------------------------------------

------------------------------------------------------------

------------------------------------------------------------

------------------------------------

>VvCYC FQ382723

------------------------------------------------------------

------------------------------------------------------------

------------------------------------------------------------

------------------------------------------------------------

------------------------------------------------------------

------------------------------------------------------------

------------------------------------------------AAGAGGGATCGG

---CACAGCAAGATAAATACTGCCCGAGGCCCCAGAGATCGGAGAATGAGATTGTCCCTC

GAAATCGCTCGAAAGTTCTTTGATCTGCAAGACATGCTAGGCTTTGATAAAGCTAGTAAA

ACTGTTGAATGGTTACTAATAAAGGCCAAATCTGCCATCAAAGAACTG------------

------------------------------------------------------------

------------------------------------------------------------

------------------------------------------------------------

---------------------------------------------------AGG---GAG

TCGAGGGAAAAGGC---AAGAGCAAGGGCAAGGGAGAGGACAAGAGAAAAG---------

------------------------------------------------------------

------------------------------------------------------------

------------------------------------------------------------

------------------------------------------------------------

------------------------------------------------------------

------------------------------------------------------------

------------------------------------

>AcCYC1a JQ772502

------------------------------------------------------------

------------------------------------------------------------

------------------------------------------------------------

------------------------------------------------------------

------------------------------------------------------------

------------------------------------------------------------

------------------------------------------------AAAAAGGATCGG

---CACAGCAAGATTTATACTGCTCGGGGTCCTAGAGATCGAAGAATGAGATTGTCCCTT

GAGGTTGCCCGTGAGTTTTTTGATCTTCAAGACATGCTGGAATTCGATAAAGCGAGTAAA

ACTGTGGAGTGGCTCTTGTCACAATCCAAGTCGGCGATCAAAGAGCTT------------

------------------------------------------------------------

------------------------------------------------------------

------------------------------------------------------------

---------------------------------------------------AGA---GAA

GCGAGGAAAAAGGC---GAGGGCGAGGGCGAGGGAGAGAACAGTAGAGAAG---------

------------------------------------------------------------

------------------------------------------------------------

------------------------------------------------------------

------------------------------------------------------------

------------------------------------------------------------

------------------------------------------------------------

------------------------------------

>PoCYC HQ599283

------------------------------------------------------------

------------------------------------------------------------

------------------------------------------------------------

------------------------------------------------------------

------------------------------------------------------------

------------------------------------------------------------

------------------------------------------------AAGATAGATCGT

---CACAGCAAGATTTTCACAGCTCAAGGTCCCCGGGACCGGAGAATGAGACTGTCCGTC

GAGATTGCCCGTCAGTTCTTTGATCTCCAAGACATGCTTGGGTTCGATAAAGCTAGTAAA

ACCATTGAGTGGCTGCTTACAAAGTCGAAGGCCGCAATCAAGGAGCTG------------

------------------------------------------------------------

------------------------------------------------------------

------------------------------------------------------------

---------------------------------------------------AGA---GAA

TCCAGGAAAAAGGC---TAGAGCACGGGCTAGGGAGAGGACAATAGAGAAG---------

------------------------------------------------------------

------------------------------------------------------------

------------------------------------------------------------

------------------------------------------------------------

------------------------------------------------------------

------------------------------------------------------------

------------------------------------

>AtBRC2 NM105554

------------------------------------------------------------

------------------------------------------------------------

------------------------------------------------------------

------------------------------------------------------------

------------------------------------------------------------

------------------------------------------------------------

------------------------------------------------AAAAGGGACAGG

---CATAGTAAGATCTGCACGGCTCAAGGTCCTAGAGACCGGAGGATGAGGCTGTCTCTT

CAGATTGCTCGCAAGTTTTTCGATCTTCAAGACATGTTGGGTTTCGACAAGGCGAGCAAG

ACGATTGAATGGCTTTTCTCCAAATCAAAGACTTCCATCAAACAACTT------------

------------------------------------------------------------

------------------------------------------------------------

------------------------------------------------------------

---------------------------------------------------AAA---GAG

TCGAGAGAGAGAGC---TAGAAAGCGAGCAAGAGAGAGAACAATGGCAAAG---------

------------------------------------------------------------

------------------------------------------------------------

------------------------------------------------------------

------------------------------------------------------------

------------------------------------------------------------

------------------------------------------------------------

------------------------------------

>AlBRC2 XM002888638

------------------------------------------------------------

------------------------------------------------------------

------------------------------------------------------------

------------------------------------------------------------

------------------------------------------------------------

------------------------------------------------------------

------------------------------------------------AAAAGGGACAGA

---CATAGTAAGATCTGCACGGCTCAAGGTCCTAGAGACCGGAGGATGAGGCTGTCTCTT

CAGATTGCTCGCAAGTTTTTCGATCTTCAAGACATGTTGGGTTTCGACAAGGCGAGCAAG

ACGATTGAATGGCTTTTCTCCAAATCAAAGGGTTCCATCAAACAACTT------------

------------------------------------------------------------

------------------------------------------------------------

------------------------------------------------------------

---------------------------------------------------AAA---GAG

TCGAGAGAGAGAGC---TAGAAAACGAGCAAGAGAGAGAACAATGGCAAAG---------

------------------------------------------------------------

------------------------------------------------------------

------------------------------------------------------------

------------------------------------------------------------

------------------------------------------------------------

------------------------------------------------------------

------------------------------------

>PtCYC XM002314850

------------------------------------------------------------

------------------------------------------------------------

------------------------------------------------------------

------------------------------------------------------------

------------------------------------------------------------

------------------------------------------------------------

------------------------------------------------AAGAAGGACAGG

---CATAGTAAGATCCATACTGCTCAAGGGCCGAGAGACCGAAGGATGAGGTTATCACTT

CAAATTGCCAGAAAGTTCTTTGATCTCCAAGACATGCTCGGCTTCGATAAAGCAAGCAAA

ACTATCGAGTGGCTCTTTACAAAATCGAAGGCCGCGATCAAGGAGCTC------------

------------------------------------------------------------

------------------------------------------------------------

------------------------------------------------------------

---------------------------------------------------AGA---GAA

TCTAGGGAGAAAGC---AAGGGCAAGGGCAAGGGATCGAACAAGAGAGAAA---------

------------------------------------------------------------

------------------------------------------------------------

------------------------------------------------------------

------------------------------------------------------------

------------------------------------------------------------

------------------------------------------------------------

------------------------------------

>RcCYC XM002524354

------------------------------------------------------------

------------------------------------------------------------

------------------------------------------------------------

------------------------------------------------------------

------------------------------------------------------------

------------------------------------------------------------

------------------------------------------------AAAAAGGACAGG

---CACAGCAAGATCTATACAGCTCAAGGGCCAAGAGACAGGAGAATGAGGTTGTCTCTT

CAAATTGCTAGGAAGTTCTTTGATCTACAAGACATGTTAGGCTTCGATAAAGCAAGCAAA

ACAATTGACTGGCTATTCACGAAATCAAAGGCAGCAATCAAGGAACTA------------

------------------------------------------------------------

------------------------------------------------------------

------------------------------------------------------------

---------------------------------------------------AGA---GAG

TCCAGAGACAAGGC---AAGAGCAAGAGCAAGGGAAAGAACAAAAGAGAAA---------

------------------------------------------------------------

------------------------------------------------------------

------------------------------------------------------------

------------------------------------------------------------

------------------------------------------------------------

------------------------------------------------------------

------------------------------------

>GhCYC6 JN190060

------------------------------------------------------------

------------------------------------------------------------

------------------------------------------------------------

------------------------------------------------------------

------------------------------------------------------------

------------------------------------------------------------

------------------------------------------------AAGAAAGACAGG

---CATAGCAAAATTCATACAGCTCAAGGTCTTCGAGACAGACGAATGAGGTTGTCTCTA

CAAATCGCACGCAAGTTTTTTGGTCTTCAAGACATGTTAGGCTTTGACAAAGCTAGTAAA

ACTATCGAGTGGCTCTTTTGCAAGTCCAAGAAGGCAATCGAAGAAGTC------------

------------------------------------------------------------

------------------------------------------------------------

------------------------------------------------------------

---------------------------------------------------AGG---GAG

TCGAGGGACAAGGC---GAGAGCAAGAGCAAGAGAGAGAACAAGAGAGAGA---------

------------------------------------------------------------

------------------------------------------------------------

------------------------------------------------------------

------------------------------------------------------------

------------------------------------------------------------

------------------------------------------------------------

------------------------------------

>GhCYC8 JN190062

------------------------------------------------------------

------------------------------------------------------------

------------------------------------------------------------

------------------------------------------------------------

------------------------------------------------------------

------------------------------------------------------------

------------------------------------------------AAGAAAGACAGG

---CACAGCAAGATACACACAGCTCAAGGTCTTAGAGACAGGAGAATGAGGTTGTCACTT

CATATTGCCCGAAAGTTTTTCGATCTTCAAGACTTGTTAGGGTTTGACAAAGCCAGCAAG

ACCATTGAGTGGCTCTTTTGCAAGTCCAATAAAGCCATCAAAGAGGT-------------

------------------------------------------------------------

------------------------------------------------------------

------------------------------------------------------------

-----------------------------------------------------G---TTA

GCAAGGGAGCAGGC---GAGAGAACGAGCAAGGGAGAGAACAAGAGAGAAA---------

------------------------------------------------------------

------------------------------------------------------------

------------------------------------------------------------

------------------------------------------------------------

------------------------------------------------------------

------------------------------------------------------------

------------------------------------

>SolBRC2a

ATGTTTCCTTCAAGCAA---TAACCATGATACTTTTTCTTATACCTCAAAAACATATCTT

GAGAGATCTTTTACATATGATCATCAAAACCCTAGTTCAAGTTCAAGACAAGAAGATAAT

CCATTTTTCTTGAACTTCCCTTCTCCATTTCTTGATCATAATGAGTCTCCCTTGAGCCAA

ATATTACCTCAAGATCATCATGTGAAAGAGGGAAATTTAACTCATCTTTCAAGTGAAACA

TCAAAGGAGGAAATGTCTATTGAGGCTAAACCATCATCAAAGAAGAGAAGTCTTAGCACA

ACGCCACGAAAGAGGACGGGG---------------------------------------

------------------------------------------------AAGAAGGATAGA

---CATAGCAAGATTTGCACTGCTCAAGGTGTAAGGGATCGAAGAGTAAGATTATCCCTT

CACATAGCGCGTAAGTTCTTTGATCTCCAAGACATGTTAGGCTTTGATAAAGCAAGTAAA

ACCATAGAATGGCTTTTCTCCAAATCCAATAATGCCATAAAAGATCTC------------

------------------------------------------------------------

TCAGAAAACACCCCACAAAAAGAATATAGTGATGGTAACAAGATTGTTATAAATAGTAAC

AATAGTTCAAGTTATGAAGGGAAGAGTGATTCCTTTATGTCTGAGTGTGAGGAGAACTCA

ATCAATGAATTAGGTAAAGATAAGGAAAAAATTATGCAAAATAATCCTCATAAAAGGGAA

TCAAGGGAAAAAGC---------AAGAGCAAGGGCTAGAACCAAAGAAAAAATGATGATC

AAAGGTCTTGAAAAAGGTAACCCTAGTAACATGTTTGATCAATTAGGATCATCAAGAAGC

AATAGTGGTTTTCTTGATCAAGATTCAAATAATAATTCTTACAACACAAGTGTTAATCAA

GAAAAAGGTTCACCACATGAAGCCAATTCTCAATCTCTAGAACATCATTTTCCAATTCAA

AATTATTTAGGTGGTGCAAGTAATTCATCAACAATAGATGTTGGAAATTGCTTCATGAGT

TTTCATGGAAATTGGGAAATCAACTATGCTCCAATGAAATCAACCAACTATAGTACTACA

TTTGCAGGTAACCCTAGCTCAATTTACTTGGCTCAACAATATCAGACATTGGATCAAGAA

AAAAATCTATCCAGCAAACATCATAGATTGGAATAA

>SlBRC2 HM921067

------------------------------------------------------------

------------------------------------------------------------

------------------------------------------------------------

------------------------------------------------------------

------------------------------------------------------------

------------------------------------------------------------

------------------------------------------------AAAAAGGATAGA

---CATAGCAAGATTTGCACAGCTCAGGGTGTGAGAGATAGAAGGATGAGATTATCCCTT

CAAATAGCGCGTAAGTTCTTCGATCTCCAAGACATGTTAGGGTTTGATAAAGCAAGTAAT

ACCATAGAATGGTTGTTTTCCAAGTCCAAGAATGCCATCAAAGAGCTC------------

------------------------------------------------------------

------------------------------------------------------------

------------------------------------------------------------

---------------------------------------------------AAAATTGGT

TCAAGTGACAAGCCAATAAGAGCAAAGACAAGGGAGAAAACCAAAGAGAAA---------

------------------------------------------------------------

------------------------------------------------------------

------------------------------------------------------------

------------------------------------------------------------

------------------------------------------------------------

------------------------------------------------------------

------------------------------------

>SolBRC2b

ATGTTTCCTAAAAGCAACATCATCCATGACCCTTTTTCCTTCACCTCACAAGAATTGCTT

AAACAATCTTATAGTACACATGATCAAAACCCTAATTCACCTTCAAAAGTAGTAGAAGAT

GAAGATCATCCATTTTTCTTGAATAACTTCTTCCCATCTCCATTTCTTGATGACCATGAA

CTCCCACTAAACCAAATATTTTCTCAAAAGCATCATCAAAAGCAAGAGGCTAGTGATAAC

CATGACATTAATCAAGCTGATCCAGACAACACAATCAAAGATAATCACAGTGTTGACAAT

TCAAGATCTACACAACTCAACACGAAAATCATGGGAGATCAGAGTTCAAATCCGGCCATA

TCATCAAAGAAGAGAAAACTAAGTGCAAAACCGCGAAGGAGGACAGGAAAAAAGGATAGA

---CATAGCAAGATTTGCACAGCTCAGGGTGTGAGAGATAGAAGGATGAGATTATCCCTT

CAAATAGCGCGTAAGTTCTTCGATCTCCAAGACATGTTAGGGTTTGATAAAGCAAGTAAT

ACCATAGAATGGTTGTTTTCCAAGTCCAAGAATGCCATCAAAGAGCTC------------

------------------------------------------------------------

------------------------------------------------------------

---------------------------------------------TCTAGAAACATCTCA

CAAGAGAGTAATAGTAGTGATCAAAACAATGATGATCATAGAAAATTGAGAAAAATTGGT

TCAAGTGACAAGCCAATAAGAGCAAAGACAAGGGAGAAAACCAAAGAGAAAATGATGATC

AAATTAGGTCACAACAAAAAGGGAAATCAAGAATTAGATGAAACAAACCCTATGAGTACT

ATTGATCCTAAATTAGGGTCAAATCCAAAATCTCTTGAACATCAATTTGCTAATGTTGGG

ATCATGGAAAGGTACTTAGGTGGTGCAAGTTATTCCTCAATAACTTCAATATTTGATTAT

GACAACAATGGTGTAATCAAAGGAAACATAGACATTTCAGACAATTGCTTCAATATGGGG

ATTCTTGAAAATTGTTCAATGACAAATGAGGTTCAAATCCCATTTTCAGGTAATAACCCT

AGCTCAATTTATTTGGATTATTCAAGGTTTCATCAATTTTAG------------------

------------------------------------

>GhCYC2 EU429303

------------------------------------------------------------

------------------------------------------------------------

------------------------------------------------------------

------------------------------------------------------------

------------------------------------------------------------

------------------------------------------------------------

------------------------------------------------AAGAAAGATCAC

---CATAGTAAGATCGACACAGCTCACGGCCCTAGAGATCGGAGAGTTAGATTGTCCATT

GATATCGCAAGAAAGTTCTTCTGTCTTCAAGATTTGCTAGGGTTTGACAAAGCAAGCAAA

ACCCTTGACTGGCTCTTCACCAAGTCAAAGCCCGCCATTGATGAGCTG------------

------------------------------------------------------------

------------------------------------------------------------

------------------------------------------------------------

---------------------------------------------------AAT---CAA

TCAAGAGCAGAGGC---TAGGGCAAGAGCTAGAGAACGAACTAAAGAGAAA---------

------------------------------------------------------------

------------------------------------------------------------

------------------------------------------------------------

------------------------------------------------------------

------------------------------------------------------------

------------------------------------------------------------

------------------------------------

>GhCYC3 EU429304

------------------------------------------------------------

------------------------------------------------------------

------------------------------------------------------------

------------------------------------------------------------

------------------------------------------------------------

------------------------------------------------------------

------------------------------------------------AAGAAAGATGGA

---CATAGTAAGATCTACACGGCCCAAGGTCCTAGGGATAGGAGGGTGAGATTGTCCATT

GAGATTGCACAAAAGTTCTTCGTTCTTCAAGACTTGCTAGGGTTCGACAAAGCAAGCAAA

ACCCTTGATTGGCTCTTTACTAAGTCCAAGACTGCAATAAGAGAGCTG------------

------------------------------------------------------------

------------------------------------------------------------

------------------------------------------------------------

---------------------------------------------------GGA---GTT

TCAAGGGCAGAAGC---AAGCGCTAGAGCTAGAGAAAGAACAAAAGAAAAA---------

------------------------------------------------------------

------------------------------------------------------------

------------------------------------------------------------

------------------------------------------------------------

------------------------------------------------------------

------------------------------------------------------------

------------------------------------

>GhCYC7 JN190061

------------------------------------------------------------

------------------------------------------------------------

------------------------------------------------------------

------------------------------------------------------------

------------------------------------------------------------

------------------------------------------------------------

------------------------------------------------AAGAAAGATCGC

---CATAGCAAGATCTTCACAGCTCAAGGTCCAAGGGATCGGAGGGTGAGATTATCCATT

GAAATTTCAAGAAAGTTTTTTGGTCTTCAAGATTTGCTAGGGTTTGATAAAGCCAGCAAA

ACCCTTGATTGGCTCTTTACGAAATCCATGACTGCCATCAAGGATTTG------------

------------------------------------------------------------

------------------------------------------------------------

------------------------------------------------------------

---------------------------------------------------AGAAACCAG

TTAAGAGCAGAGGC---GAGAGCGAGAGCTAGGGAACGAACAATTGAAAAA---------

------------------------------------------------------------

------------------------------------------------------------

------------------------------------------------------------

------------------------------------------------------------

------------------------------------------------------------

------------------------------------------------------------

------------------------------------

>GhCYC4 EU429305

------------------------------------------------------------

------------------------------------------------------------

------------------------------------------------------------

------------------------------------------------------------

------------------------------------------------------------

------------------------------------------------------------

------------------------------------------------AAGAAAGATGGG

---CATAGTAAGATCTACACAGCCGGAGGCCCCAGGGATCGGAGGGTGAGATTATCCATT

GGAATCGCAAAAAAGTTCTTCTGTCTCCAAGATCTGCTAGGGTTTGACAAAGCAAGCAAA

ACCCTAGATTGGCTCTTTACCAAGTCCAAGACAGCGATTAAGGATTTG------------

------------------------------------------------------------

------------------------------------------------------------

------------------------------------------------------------

---------------------------------------------------GAC---CAA

TCAAGGGCTGAGGC---AAGAGCGAGAGCTAGAGAAAGAACAAGAGAAAAA---------

------------------------------------------------------------

------------------------------------------------------------

------------------------------------------------------------

------------------------------------------------------------

------------------------------------------------------------

------------------------------------------------------------

------------------------------------

>GhCYC9 JN190063

------------------------------------------------------------

------------------------------------------------------------

------------------------------------------------------------

------------------------------------------------------------

------------------------------------------------------------

------------------------------------------------------------

------------------------------------------------AAGAAAGATGGG

---CGTAGCAAGATCTACACAGCCGGAGGCCCTAGGGATCGGAGGGTCAGATTGTCAATT

GAAATCGCAAGAAAGTTCTTCTGTCTTCAAGATTTGCTAGGGTTTGATAAACCTAGCAAA

ACCCTTGATTGGCTCTTCGCTAAGTCCAAGACAGCGATTAAGGATTTG------------

------------------------------------------------------------

------------------------------------------------------------

------------------------------------------------------------

---------------------------------------------------AGAGACCAG

TCAAGGGCTGAAGC---AAGAGCGAGAGCTAGAGAAAGAACAAGAGAAAAA---------

------------------------------------------------------------

------------------------------------------------------------

------------------------------------------------------------

------------------------------------------------------------

------------------------------------------------------------

------------------------------------------------------------

------------------------------------

>GhCYC5 JN190059

------------------------------------------------------------

------------------------------------------------------------

------------------------------------------------------------

------------------------------------------------------------

------------------------------------------------------------

------------------------------------------------------------

------------------------------------------------AAGAAAGATGGG

---CATAGTAAAATCTATACGGCTCAAGGCCCTAGAGATCGGAGAGTGAGATTATCCATT

GAGATTGCCCAAAAGTTTTTTGTGCTTCAAGACCTGCTAGGGTTTGACAAGGCAAGCAAA

ACCCTTGATTGGTTGTTTACCAAGCCCAAGACGGCGATTAAGGAATTG------------

------------------------------------------------------------

------------------------------------------------------------

------------------------------------------------------------

---------------------------------------------------GAC---CTG

TCAAGGGCAGAGGC---AAGAGCAAGAGCAAGGGAAAGGACAAAAGAAAAA---------

------------------------------------------------------------

------------------------------------------------------------

------------------------------------------------------------

------------------------------------------------------------

------------------------------------------------------------

------------------------------------------------------------

------------------------------------

>HaCYC2c EU088370

------------------------------------------------------------

------------------------------------------------------------

------------------------------------------------------------

------------------------------------------------------------

------------------------------------------------------------

------------------------------------------------------------

------------------------------------------------AAAAAAGATGGG

---CATAGTAAAATCTATACCGCACAAGGCCCGAGAGATCGGAGGGTGAGATTGTCCATT

GACATTGCAAGAAAGTTTTTTGTGCTTCAAGATTTGCTAGGTTTTGACAAAGCAAGCAAA

ACCCTTGATTGGCTCTTTACCAAGTCCAAGAAGGCGATTAAGGAGTTG------------

------------------------------------------------------------

------------------------------------------------------------

------------------------------------------------------------

---------------------------------------------------AGAGGCCAG

TCAAGGGCAGAGGC---AAGAGCAAGGGCTAGGGAGAGAACAAAACAAAAA---------

------------------------------------------------------------

------------------------------------------------------------

------------------------------------------------------------

------------------------------------------------------------

------------------------------------------------------------

------------------------------------------------------------

------------------------------------

>SolCYC1

ATGTTCCCTTTTGGAAACAGTAGTAATGGTGGGAACCCTATTCTTCACTCCTCTTTTCTT

AACAACCAAATACTTCTTCATCAACATGACCTTCCTACTCATCATCATTACTTAGCAGCC

GCAAATGGTCACTCAATCGACT--------------------------------------

------------------------------------------------------------

------------------------------------------------------------

------------------------------------------CGTATGCAACTAACAATG

TCGCCATCAACAACAAGAGTAA----------------AAAACAAGTGAAAAAGGATCGG

---CACACTAAGATTTTGACATCACAAGGGCATAGGGATCGGAGGGTGAGGTTGTCGATA

GGGGTTGCTCGTAAGTTCTTTGATTTGCAAGACATGCTTGGTTACGATAAACCAAGTAAA

ACCCTTGATTGGCTATTCACCAAGTCTAAATTAGCCATTGAAGATCTC------------

------------------------------------------------------------

ATCAACGATGTGTCAAAGAAGAGTACTCCTTTATCTATTCATAATA--------------

----------------------------------------ATAATAATAATAACAATTCG

GAATGTGATGAGGATATGATTGTTCCTCTTGCAAAAAAAGCAAAGCAAGAGAGA---GAC

TCAAGGGCAAAGGC---TAGAGCAAGAGCTAGAGAAAGAACTATTAAGAAAATTTGGACC

CAAATTGCTCCGAATAGAGAAGCTACAGCTTCACATTACAATAATTCGACACGAAATTGG

AATCATGACGATGTTAATCCAACAATCATGAGTTCCATGGATGCATCTACCATTTGTTGT

ACCTCATTACCGATAGGTAAAAAACTAAAATACTACTTATAA------------------

------------------------------------------------------------

------------------------------------------------------------

------------------------------------------------------------

------------------------------------

>AcCYC2 JQ772501

------------------------------------------------------------

------------------------------------------------------------

------------------------------------------------------------

------------------------------------------------------------

------------------------------------------------------------

------------------------------------------------------------

------------------------------------------------AAGAAGGACAGG

---CACAGCAAGATATACACCGCACAGGGCCTCCGAGACCGGAGGGTGAGGCTCTCGATT

GAGATCTCGCGCAGGTTCTTCGACCTCCAGGACATGCTAGGGTTCGACAAGGCAAGCAAA

ACCCTAGAGTGGCTCCTTTCGAAATCGAGGAAGGCCATCAAGGATCTG------------

------------------------------------------------------------

------------------------------------------------------------

------------------------------------------------------------

---------------------------------------------------GAA---GAG

TCTAGGGCAAAGGC---AAGGGCTAGGGCAAGGGCTAGGACCAGAGCAAAA---------

------------------------------------------------------------

------------------------------------------------------------

------------------------------------------------------------

------------------------------------------------------------

------------------------------------------------------------

------------------------------------------------------------

------------------------------------

>RcCYC2 XM002521155

------------------------------------------------------------

------------------------------------------------------------

------------------------------------------------------------

------------------------------------------------------------

------------------------------------------------------------

------------------------------------------------------------

------------------------------------------------AAGAAACATAGG

---CACAGCAAGATTTGCACAGCTCAAGGTTTAAGAGATCGGAGAGTGAGATTGTCCATC

GAAATTGCTCGAAAGTTCTTTGATCTTCAGGATTTGCTAGGGTTTGATAAAGCAAGTAAA

ACCCTAGAATGGTTGTTGTCCAAGTCAAGAAAAGCCATTAAAGCGCTA------------

------------------------------------------------------------

------------------------------------------------------------

------------------------------------------------------------

---------------------------------------------------AAA---GAG

TCAAGAGCAAAAGC---AAGAGAAAGAGCAAGAGAAAGAACTAGAGTTAAA---------

------------------------------------------------------------

------------------------------------------------------------

------------------------------------------------------------

------------------------------------------------------------

------------------------------------------------------------

------------------------------------------------------------

------------------------------------

>MtCYC XM003637000

------------------------------------------------------------

------------------------------------------------------------

------------------------------------------------------------

------------------------------------------------------------

------------------------------------------------------------

------------------------------------------------------------

------------------------------------------------AAGAAAGACCGA

---CACAGCAAGATTTACACTTCTCAAGGTTTGAGAGATCGAAGAGTGAGGCTTTCGATT

GAGATCGCTCGAAAGTTCTTCGATCTTCAAGACATGTTAGGGTTTGACAAAGCTAGCAAC

ACACTTGATTGGCTTTTCACAAAATCTAAGAAAGCAATTAAGGATCTA------------

------------------------------------------------------------

------------------------------------------------------------

------------------------------------------------------------

---------------------------------------------------AAG---GAG

TCAAGGGAAAAAGC---AAGAGCAAGAGCAAGGGAAAGAACTAGTAACAAG---------

------------------------------------------------------------

------------------------------------------------------------

------------------------------------------------------------

------------------------------------------------------------

------------------------------------------------------------

------------------------------------------------------------

------------------------------------

>CpCYC AY225825

------------------------------------------------------------

------------------------------------------------------------

------------------------------------------------------------

------------------------------------------------------------

------------------------------------------------------------

------------------------------------------------------------

------------------------------------------------AAGAAAGATAGG

---CACAGCAAGATTTACACCTCCCAGGGCTTGAGGGACCGCAGGGTGAGGTTGTCCATT

GAGATCGCCCGCAAGTTCTTTGATCTACAAGACATGCTAGGGTTTGACAAAGCCAGTAAC

ACTCTTGAGTGGCTCTTCAACAAGTCCAAGAAAGCAATTAAAGATCTA------------

------------------------------------------------------------

------------------------------------------------------------

------------------------------------------------------------

---------------------------------------------------AAG---GAG

TCCAGAGAAAAAGC---AAGAGCAAGAGCAAGGGAAAGGACTAGTAACAAG---------

------------------------------------------------------------

------------------------------------------------------------

------------------------------------------------------------

------------------------------------------------------------

------------------------------------------------------------

------------------------------------------------------------

------------------------------------

>LnCYC AY382155

------------------------------------------------------------

------------------------------------------------------------

------------------------------------------------------------

------------------------------------------------------------

------------------------------------------------------------

------------------------------------------------------------

------------------------------------------------AAAAAGGATAGG

---CACAGCAAGATTTACACCTCTCAGGGCTTGAGGGATCGGAGGGTGAGGCTTTCGATT

GAGATCGCGCGAAAGTTCTTCGATCTACAAGACATGCTAGGGTTTGACAAAGCAAGCAAC

ACCCTTGAGTGGCTCTTCAACAAGTCCAAGAGAGCAATTAAGGACCTA------------

------------------------------------------------------------

------------------------------------------------------------

------------------------------------------------------------

---------------------------------------------------AAA---GAG

TCCAGGGAAAAAGC---AAGAGCAAGAGCAAGAGAAAGAACTAGTAACAAG---------

------------------------------------------------------------

------------------------------------------------------------

------------------------------------------------------------

------------------------------------------------------------

------------------------------------------------------------

------------------------------------------------------------

------------------------------------
